# Host manipulation in the face of environmental changes: Ecological consequences

**DOI:** 10.1016/j.ijppaw.2015.08.001

**Published:** 2015-08-25

**Authors:** Sophie Labaude, Thierry Rigaud, Frank Cézilly

**Affiliations:** Université de Bourgogne Franche-Comté, UMR CNRS 6282 Biogéosciences, Dijon, France

**Keywords:** Ecosystems, Environment, Global changes, Host manipulation, Host–parasite interactions

## Abstract

Several parasite species, particularly those having complex life-cycles, are known to induce phenotypic alterations in their hosts. Most often, such alterations appear to increase the fitness of the parasites at the expense of that of their hosts, a phenomenon known as “host manipulation”. Host manipulation can have important consequences, ranging from host population dynamics to ecosystem engineering. So far, the importance of environmental changes for host manipulation has received little attention. However, because manipulative parasites are embedded in complex systems, with many interacting components, changes in the environment are likely to affect those systems in various ways. Here, after reviewing the ecological importance of manipulative parasites, we consider potential causes and consequences of changes in host manipulation by parasites driven by environmental modifications. We show that such consequences can extend to trophic networks and population dynamics within communities, and alter the ecological role of manipulative parasites such as their ecosystem engineering. We suggest that taking them into account could improve the accuracy of predictions regarding the effects of global change. We also propose several directions for future studies.

## Highlights

• Environmental changes can affect ecosystems in various ways.
• Manipulative parasites are known to play numerous roles within ecosystems.
• However, the effects of environmental changes on manipulation has been overlooked.
• We review those effects and their potential consequences on larger scales.
• We conclude with suggestions on the direction of future studies.

## Graphical abstract

**Figure:**
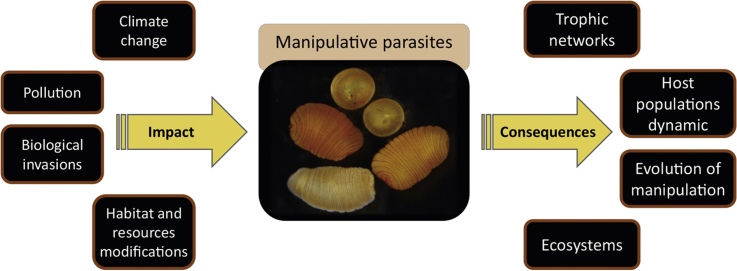

## 1 Introduction

Understanding the consequences of environmental changes has become a major challenge in recent years in many fields of science. Parasitology is among the most sensitive topics regarding the effects of global changes, since accurate predictions about the expansion of parasites and their hosts might be essential to take appropriate measures to prevent epidemic diseases. Moreover, an increasing number of reviews have highlighted the potential impact of climate change on parasitism (e.g. MacLeod and Poulin, 2012, Marcogliese, 2001, Morley and Lewis, 2014). As a result, the number of theoretical models providing simulations about the future geographical range of parasites and their vectors is increasing too. However, most predictive parasitological studies have been limited to vector-borne diseases affecting either humans, livestock, or domestic animals (Genchi et al., 2009, Giles et al., 2014, Moore et al., 2012, Mordecai et al., 2013, Paaijmans et al., 2010, Stensgaard et al., 2013, Sternberg and Thomas, 2014, White et al., 2003), with noticeable exceptions such as blood parasites in wild birds (Fuller et al., 2012, Loiseau et al., 2013).

Parasitic organisms altogether might represent close to half of all biodiversity (Dobson et al., 2008, Poulin and Morand, 2000). Apart from causing diseases, there is increasing evidence that they can play pivotal roles in ecosystems (Thomas et al., 1997; Hatcher et al., 2012). In particular, many parasites are able to alter their hosts’ phenotypes, with far-reaching consequences for, for instance, population dynamics or the persistence of species in ecosystems (Lefèvre et al., 2009).

Parasites that are able to manipulate their hosts are very diverse, ranging from viruses (Ingwell et al., 2012) and bacteria (Werren et al., 2008) to many eukaryote organisms, including animals such as cestodes, trematodes, or acanthocephalans (Poulin and Thomas, 1999). The number of hosts susceptible to be manipulated by parasites is also wide, including both vertebrate and invertebrate species (Poulin and Thomas, 1999), and even plants (Ingwell et al., 2012). Interestingly, the inventory of manipulative parasites also includes medically and veterinary important species that are already well studied (Hurd, 2003, Lagrue and Poulin, 2010), such as parasites causing malaria (Koella et al., 1998), toxoplasmosis (Berdoy et al., 2000), or rabies (Klein, 2003). However, even though the manipulative abilities of those parasites could have implications for epidemiology and pathology (Lagrue and Poulin, 2010), epidemiologic models tend to completely ignore them.

Similarly, despite the importance of host manipulation by parasites for ecosystems and health, the effects of environmental changes on their ecological roles are largely ignored. After emphasizing the ecological importance of manipulative parasites, we show here that environmental changes can interact with them in many different ways, leading to consequences that deserve more attention, especially in the area of conservation, in order to make accurate predictions regarding the effects of global change.

## 2 Ecological importance of host manipulation by parasites

Parasites are widely recognized to have numerous effects on communities and ecosystems, in particular through density-dependent pathogenic effects on their hosts (Hatcher et al., 2012). For instance, differential host susceptibility and tolerance can reverse the outcome of competition, when the fitness of the superior competitor is more impaired by parasitic infection than that of other host species. The presence of parasites might then lead to the coexistence of several species that would otherwise exclude each other. Moreover, parasites influence the organization of communities and, through that, play such an important role in the stability of ecosystems that they have been proposed to serve as a proxy of their quality (Hudson et al., 2006). On the other hand, parasites can also have negative effects on biodiversity, such as causing local extinctions (McCallum and Dobson, 1995).

An important aspect is that all parasites are embedded in large food webs. In particular, parasites with complex life-cycles have the potential to impact several host species in succession, making their global impact (see below) even more consequent. Some of those parasites are able to induce phenotypic modifications in their intermediate hosts, which are believed to be more than simple pathological effects. Through host manipulation, parasites are thought to enhance their own fitness, in particular by increasing their probability of transmission from one host to another, at the expense of that of their hosts (Thomas et al., 2005). Many theoretical as well as empirical studies have highlighted that this phenomenon, along with more classic pathogenic effects, can have profound ecological impacts on a large scale, ranging from host populations to ecosystems (Lefèvre et al., 2009). Although manipulative parasites can affect ecosystems in diverse ways, three major effects can be distinguished: the impact of parasites on food webs, their influence on the population dynamics of host species, and their impact on habitats.

### 2.1 Impact on food webs

Trophically-transmitted parasites often manipulate their intermediate hosts in ways that increase their probability of being predated by definitive hosts. For instance, killifish (*Fundulus parvipinnis*) parasitized by the trematode *Euhaplorchis californiensis* are up to 31 times more susceptible to predation than uninfected individuals (Lafferty and Morris, 1996). The effect on the energy flow is even more substantial considering that the increased vulnerability to predation induced by parasites is often not restricted to suitable hosts (Kaldonski et al., 2008, Seppälä et al., 2008b), leading to a higher predation by other species, as illustrated by cockles (*Austrovenus stutchburyi*) being exploited as intermediate hosts by trematode parasites. Infected cockles typically remain lying on the sediment surface (Thomas and Poulin, 1998), where they are more conspicuous to birds that serve as a definitive host for trematodes. However doing so, infected cockles also become more vulnerable to predation by fish which constitute 'dead-end' predators for parasites (Mouritsen and Poulin, 2003).

Manipulative parasites can also create new trophic interactions. One of the most spectacular examples comes from nematomorph parasites (*Gordionus* spp.), which induce their terrestrial insect hosts into jumping in the water (a crucial stage in the life cycle of the parasite; Sato et al., 2011). Empirical evidence shows that manipulated insects represent a new and substantial energy intake for fish (Sato et al., 2011), with the interesting consequence of decreasing fish predation on benthic invertebrate communities, thus leading to subsequent decrease in algae biomass, and, ultimately, to a reorganization of the whole ecosystem (Sato et al., 2012).

Another impact of parasites on food webs, though not necessarily restricted to manipulative ones, lies in the alteration of the functional role of their hosts. For instance, several acanthocephalan parasites are known to alter the feeding ecology of their intermediate hosts, decreasing predation rate in amphipods (Fielding et al., 2003) or reducing the consumption of detritus in isopods (Hernandez and Sukhdeo, 2008). Such alterations can have substantial effects within ecosystems, especially when modified host species play important functional roles (Hernandez and Sukhdeo, 2008).

### 2.2 Impact on population dynamics

Host modifications induced by manipulative parasites are likely to alter hosts population dynamics and structure. For instance, the trematode *Gynaecotyla adunca* alters the vertical distribution of its snail host on sandbars (Curtis, 1987). Several gammarid species infected by acanthocephalan parasites present altered geotactic or phototactic preferences (Bauer et al., 2005, Bauer et al., 2000, Haine et al., 2005), supposed to drive them to areas where they are more exposed to predators. By altering both the behavior and morphology of their hosts, parasites can then lead them to occupy new ecological niches (Miura et al., 2006, Ponton et al., 2005). Along with effects on individual distribution, other phenotypic alterations induced by manipulative parasites are likely to induce ecological segregation, through dividing the host population into two sub-units consisting of infected vs. uninfected individuals, each of them having its own properties (Lefèvre et al., 2009).

Manipulative parasites are also likely to modify predator-prey dynamics. Evidence from mathematical modelling (Fenton and Rands, 2006) suggests that manipulation can influence both predators' and prey's abundance, and induce oscillations in their population densities that are likely to have consequences on the dynamics of other species within the ecosystem. Accordingly, Lafferty and Kuris (2012) suggested that the parasite *Echinococcus granulosus* might be responsible for the persistence of moose and wolves on Isle Royale. Indeed, recordings suggest that infection with *E. granulosus* increases moose vulnerability to wolves (Joly and Messier, 2004). As suggested by another mathematical model (Hadeler and Freedman, 1989), the parasite might be essential for wolves to be able to feed on moose, and to persist in the ecosystem. The presence of the parasite and its interaction with moose and wolves might actually prevent the demographic explosion of moose populations, which would lead to over-grazing followed by starvation, as was observed before colonization by wolves (Lafferty and Kuris, 2012).

Similarly, manipulative parasites can drive competition between hosts. In the same way that non-manipulative parasites can affect closely-related host species with different susceptibility and tolerance to infection, host species can also present different susceptibility to manipulation. Hatcher et al. (2014) used a mathematical model to show that parasite manipulation can change the outcome of the competition between two hosts showing mutual predation, and determine whether the two host species can coexist or not. In addition, some studies have shown that parasites do not always manipulate closely-related host species to the same extent (Bauer et al., 2000, Thomas et al., 1995). For instance, amphipods *Gammarus pulex* infected by the acanthocephalan *Pomphorhynchus laevis* show reversed phototaxis, while that of infected *Gammarus roeseli* remains unaltered (Bauer et al., 2000).

### 2.3 Impact on habitats

By modifying the phenotypes of their hosts, manipulative parasites may create new habitats for other species, or change habitats' parameters, endorsing the role of ecosystem engineers (Thomas et al., 1999). When infected by the parasite *Sacculina carcini*, the green crab, *Carcinus maenas*, stops molting (O'Brien and van Wyk, 1985). Its cuticle then becomes a permanent substrate on which an epibiont community can develop (Mouritsen and Jensen, 2006, Thomas et al., 1999). Another illustration comes from cockles (see paragraph 2.1.) infected by trematode parasites. Parasitized individuals, which are unable to burrow in the sand, also become a substrate with new properties for epibionts to colonize. Thomas et al. (1998) showed that the presence of parasites can then facilitate the coexistence of two epibionts, anemones and limpets, by providing the limpets with a new substrate unsuitable for anemones due to their vulnerability to desiccation, thus preventing them from predating upon limpets. Moreover, Mouritsen and Poulin (2005) put forward that biodiversity is higher on mudflats when those parasites are present, an observation that could be explained by the cockles' impaired bioturbation potential.

## 3 How environmental changes can alter the roles of manipulative parasites

**Fig. 1:**
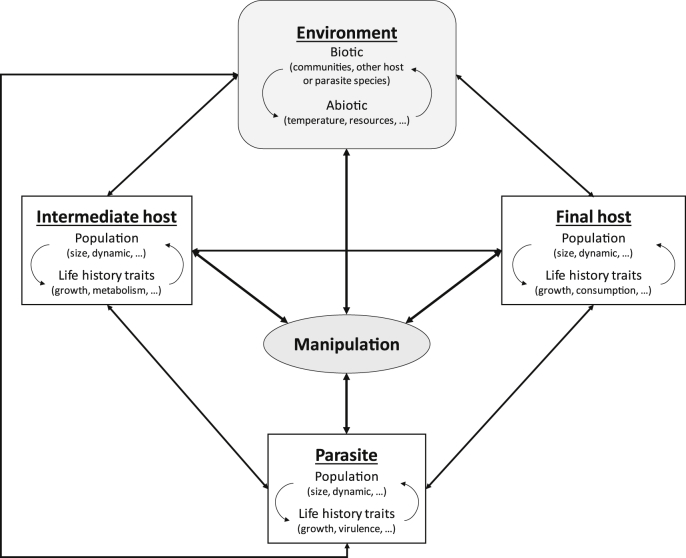
Schematic representation of all the interacting factors in a system involving parasite manipulation. The intensity of host manipulation induced by parasites is likely to be influenced by a variety of parameters concerning the parasites, their hosts and environmental properties. In return, manipulation can also have an impact on those parameters. Moreover, all components in the systems also interact with each other.

Parasite manipulation results from complex interactions between properties of parasites, properties of their hosts, and many biotic and abiotic environmental factors (Fig. 1). It appears therefore very plausible that any environmental change might affect not only manipulation itself, but also its consequences. Considering the effects of parasite manipulation on a large scale, those consequences might in turn induce new environmental changes or modify their intensity, thus altering the role of parasites within ecosystems. To emphasize the complexity behind all the interacting components of systems involving parasite manipulation, illustrated in Fig. 1, we provide here a few examples about the outcome of the interaction between manipulative parasites and several environmental modifications of major concern.

### 3.1 Climate change

**Fig. 2:**
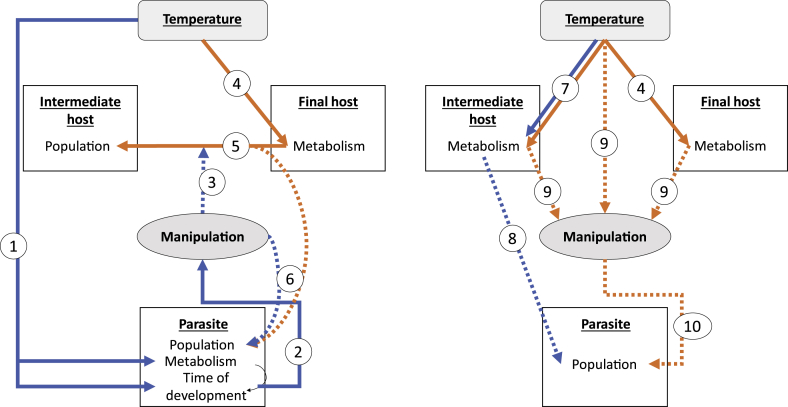
Examples of the impacts of temperature on a system of gammarid species infected by acanthocephalan parasites. Final host varies depending on parasite species (either a fish or a bird). Solid lines represent assumption supported by studies, while dotted lines are expectations that remain to be investigated. In this system, (1) the temperature widely influences the time of development or parasites within the intermediate hosts, which is likely to be driven by the metabolic rate of parasites (Tokeson and Holmes, 1982). Several studies suggested that (2) the time of development of parasites is linked to the intensity of their manipulation (Franceschi et al., 2010b, Franceschi et al., 2008), which in turn might (3) influence the increase of predation rate between the final host and the intermediate host. (4) Temperature is also likely to influence the final host metabolism (Byström et al., 2006), (5) influencing its predation rate (Byström et al., 2006). Altogether, (6) modifications in manipulation and predation rates are likely to induce changes in parasites' population. Meanwhile, (7) temperature also affects the metabolism of gammarid hosts (Issartel et al., 2005), inducing changes in their food consumption (Pellan et al., 2015). (8) Given that infection depends on food consumption, the risk of infection might vary accordingly, affecting parasites' population. Although its direct effect has not been investigated yet, (9) temperature is also likely to alter the intensity of manipulation, for instance through its effect on hosts' metabolism and activity, and therefore (10) secondarily impact parasite population dynamic.

Temperature is one of the most important abiotic factors affecting parasites’ biology (see Morley and Lewis, 2014, Morley, 2011, Thomas and Blanford, 2003 and references therein). When focusing on parasite manipulation, it is important to take into account that modifications induced by environmental factors on the ways parasites alter their hosts are likely to be indirect. Indeed, the intensity of parasitic manipulation is dependent on many parameters intrinsic to the physiology, morphology or population dynamic of both hosts and parasites (reviewed in Table 1). Any environmental factor affecting those parameters is then susceptible to also have effects on the extent of host modifications induced by parasites. Acanthocephalan parasites and their amphipod intermediate hosts constitute one of the most studied host–parasite systems in the word of parasite manipulation (Cézilly et al., 2013). Various studies have shown that several traits in both hosts and parasites can be important to explain variation observed in the intensity of manipulation at the intraspecific level (Table 1). Interestingly, many of those traits appear to be sensitive to temperature, as well as to other environmental factors (Fig. 2). For instance, several studies suggest that the time taken by one parasite to develop in its intermediate host could subsequently affect the intensity of behavioral alterations in that host (Franceschi et al., 2010b, Franceschi et al., 2008). As for many other parasites, the development time of acanthocephalans is largely influenced by temperature (Tokeson and Holmes, 1982), which thus can indirectly drive the intensity or timing of manipulation.

Climate-mediated physiological stress can have substantial effects on host immunity, thus increasing host susceptibility to infection (Cheng et al., 2005, Dittmar et al., 2013). Beyond an increase in the number of infected hosts, the intensity of manipulation may also depend upon host immuno-competence (Adamo, 2002). Therefore, climate-mediated stress may lead to widely infected and manipulated populations. On the other hand, some manipulative parasites have been shown to suppress the immune response of their hosts (Cornet et al., 2009), a phenomenon that could increase host susceptibility to manipulation, but also to infection by other parasites (Cornet and Sorci, 2010). The cumulative effects of both parasite immune-suppression and climate-mediated stress have not been investigated yet, but the combination of the two phenomena may ultimately increase host mortality, with potential consequences for both host and parasite population dynamics.

Several manipulative parasites also present seasonal variations, not only in their prevalence, but also in the intensity of their manipulation. For instance, some acanthocephalan parasites induce a stronger change in refuge use by their isopod hosts during spring, compared to summer or fall (Benesh et al., 2009a). Benesh et al. (2009a) suggested that seasonal variations in isopod behavioral alterations could result from a manipulation strategy adjusted to seasonal variation in the diet of definitive hosts. Regardless of whether seasonal modifications in manipulation are adaptive or not, temperature changes are very likely to alter such seasonality through their influence on both host and parasite ecology. For instance, a spatial overlap between intermediate and definitive hosts might appear only during a short period of time (Marcogliese, 2001). Under such circumstances, one would expect parasite's manipulative efforts to have been tuned by natural selection to coincide with this period, in order to maximize transmission. However, rapid changes in temperatures leading to modifications in the spatial distribution of both hosts and parasites may eventually result in the peak of manipulative efforts occurring at the wrong time.

Direct effects of temperature on host manipulation are poorly known. Considering that the behavior of uninfected individuals can be dependent upon temperature, and knowing that temperature affects both host and parasite metabolism (see for example Le Lann et al., 2014; where the behavior and physiology of both aphid hosts and their parasitoids are altered by temperature in different degrees), there is every reason to believe that temperature could affect the intensity of host modifications induced by parasites. In addition, parasite manipulation can directly involve behaviors related to temperature. For instance, Macnab and Barber (2012) showed that plerocercoid parasites induce a preference for warmer temperatures in their fish host, a result also found in snails infected by a trematode parasite (Bates et al., 2011). As it is the case for many other altered host traits, such an attraction can lead to a spatial segregation between infected and uninfected individuals. However, as the ambient temperature reaches the temperature preferred by infected individuals, this dichotomy would disappear, along with its potential environmental effects (see paragraph 2.3).

In oceans, the rise of CO_2_ not only induces an increase in temperature, but is also accompanied by a decrease of pH, a phenomenon known as ocean acidification (Feely et al., 2004). Ocean acidification induces deep biological negative consequences, such as decreased calcification rates in phytoplankton, corals and mollusks (Feely et al., 2004), but also alterations in metabolism, growth or survival in various invertebrate larvae (e.g. Bechmann et al., 2011). By analogy, similar negative effects have been suspected in parasites, particularly those with free-stage larvae (MacLeod and Poulin, 2012), and a recent study showed that exposure to experimentally acidified water reduces survival and longevity in cercariae and metacercariae of four species of marine trematodes (MacLeod and Poulin, 2015). However, another study showed that the immune response of the mussel, *Mytilus edulis*, was more affected by modifications in temperature than in pH, although both a high temperature and a decrease in pH changed the abundance and diversity of pathogens (Mackenzie et al., 2014). Indirect effects of ocean acidification on parasite manipulation can be expected, through such negative effects on hosts and parasites (Fig. 1), and could, like other stressors, destabilize trophic interactions (MacLeod and Poulin, 2012). However, the direct effect of ocean acidification on manipulation is unknown, and remains to be investigated.

### 3.2 Changes in community composition: biological invasions

The introduction of non-native species in new areas is often associated with the globalization of human transportation around the word, but also with alterations in habitat parameters, that make them suitable for non-native species. Biological invasions represent a major cause of biodiversity loss, and often induce profound changes in native communities' structure, leading to new environmental modifications (Molnar et al., 2008). The invasion success of an exotic species in a new area relies on many factors, including properties of the new ecosystem as well as properties of the invading species. There is increasing evidence that parasites may play an important role in the successful establishment of invasive species (Dunn et al., 2012). Interestingly, manipulative parasites have received much attention from scientists in relation to biological invasions.

There are many ways in which manipulative parasites can influence invasion success. First, following the “enemy release hypothesis”, species might escape their parasites when invading a new area (Torchin and Mitchell, 2004, Torchin et al., 2002). This phenomenon might, among other reasons, result from the fact that the invasion process is initiated by a small number of individuals, thus reducing the probability that they bring with them the whole community of parasite species from their native range. Moreover, manipulative parasites often present complex life-cycles, and are thus sensitive to the absence of any obligatory host in the new ecosystem. Torchin et al. (2005) found that while a native mud snail was infected by ten native trematode parasites, an introduced sympatric mud snail only harbored one introduced trematode. This “enemy release” directly leads to “parasite manipulation release”, which is likely to have consequences. For instance, the predation facilitation induced by some parasites is supposed to negatively impact the population dynamics of their hosts. Conversely, an absence of parasites might then lead to an explosion of the host population (as suggested above in the case of moose and wolves; see paragraph 2.2).

Parasites can also have indirect effects by affecting the competitive interactions between native and invasive closely-related host species, through differential effects on each host species (Hudson and Greenman, 1998; see paragraph 2.3). Mediated competition is often highlighted in the case of parasites causing a higher mortality due to pathogenic effects in one of the competitive host species (Dunn et al., 2012). Apart from pathogenic effects, host mortality can also be driven by the consequences of manipulation, especially when parasites alter the behavior of their intermediate hosts in ways that increase their probability of being predated by definitive hosts. In many French rivers, the native amphipod *G. pulex* has to face competition from its closely-related invader, *G. roeseli* (Karaman, 1977). Although both species can be infected by the acanthocephalan *P. laevis*, only the native species shows a reversed phototactic behavior when infected (Bauer et al., 2000). The same result has been found in the Irish native amphipod *G. duebeni celticus*, whose phototaxis is altered by the acanthocephalan *Polymorphus minutus*, while that of its invasive rival *Gammarus tigrinus* is not (MacNeil et al., 2003a, Macneil et al., 2003b). In both cases, only the native species has to face an increase in predation by fish when infected, which is likely to facilitate the invasion by the congeneric rival species (Lagrue et al., 2007). However, other altered behaviors may influence the competition between native and exotic rivals. For instance, the Irish amphipod *G. d. celticus* is being replaced by the introduced *G. pulex*, which induces numerous changes in freshwater macroinvertebrate communities (Kelly et al., 2006). Dick et al. (2010) reported that *G. pulex* harboring the acanthocephalan *Echinorhynchus truttae* have a higher predatory rate, consuming significantly more preys than uninfected individuals. Together with a higher parasitic prevalence compared to the native species (Dick et al., 2010), this functional response could give a competitive advantage to the invasive species. Conversely, Sargent et al. (2014) found that parasites *Microphallus* spp. reduce the foraging behavior of the invasive crayfish species *Orconectes rusticus*, potentially affecting its invasion success.

Competition between native and exotic species can be more direct, particularly when predation occurs between them. Manipulative parasites have the potential to drive the outcome of such a competition, as has been shown by Hatcher et al. (2014) (see paragraph 2.2). The replacement of Irish *G. d. celticus* amphipods by *G. pulex* (see above) can be partly explained by mutual predation biased in favor of the invader. However, infection with the acanthocephalan *E. truttae* reduces the predatory impact of the exotic species, thus potentially slowing down the invasion process (MacNeil et al., 2003a, Macneil et al., 2003b). This example also highlights the complexity of the impact of parasites: being infected can be both a disadvantage (lowered ability to predate upon the competitor species) and an advantage for the invasive species (modification of the functional response, see above). In the field, the impact of parasites on the competitive abilities of their hosts can be deduced from spatial variation in co-occurrence. For instance, the amphipod *Crangonyx pseudogracilis* co-occurs with *G. pulex* more frequently when the latter is parasitized by *P. minutus*, a phenomenon that can be explained by a reduced predation rate on *C. pseudogracilis* by parasitized *G. pulex* (Macneil and Dick, 2011).

Another aspect of biological invasions concerns the introduction of new parasites within an ecosystem. In particular, invasive species can bring new parasites with them, which are also likely to interfere with the invasion process. Bacela-Spychalska et al. (2014) reported that the microsporidian *Cucumispora dikerogammari*, which dispersed together with its invasive host *Dikerogammarus villosus*, is likely to decrease its host's predatory pressure on communities through altered behavior. Moreover, the arrival of new parasite species, which might be able to affect both invasive and local host species, may increase the size of the infra-community of parasites. Many hosts would then harbor several parasites with different interests in terms of transmission, either because they target different species as final hosts or because they differ in developmental stage, and, hence, infectivity to final hosts. One of the consequences of such multi-infections, apart from increased immunological and energetic costs for the host, would be a modification of the parasite-induced alterations following a competition for manipulation inside the host (“sabotage” hypothesis, Hafer and Milinski, 2015), and thus a modification of the effects of manipulation on population dynamics (see Table 1 for examples).

Finally, even though most of the studies concerning the impact of parasites in invasions focused on the effects on invasive and native host species, it is important to keep in mind that many non-host species interact with them. Consequences might first emerge at the scale of the whole ecosystem if invasive species or their native competitors are key species, as is the case of many gammarid species (Kelly et al., 2002). In addition, in the case of invasions driven by parasites through their effects on predation facilitation, other predator species might benefit from the arrival of invasive hosts, as a new source of food. As illustrated by the case of nematomorph parasites (see paragraph 2.1), the introduction of new food resources in food webs can have large consequences on many parameters of an ecosystem.

### 3.3 Pollution

Human activities are responsible for the release of more and more pollutants in the environment, especially in freshwater ecosystems (Loos et al., 2009). Toxic chemicals could influence parasite manipulation in various ways, although the interaction between pollution and parasite manipulation itself has received very little attention from scientists (Thomas et al., 2011). As discussed earlier with the effects of climate, pollution can, in the same way, impact host or parasite traits, which could in turn have consequences on the extent of manipulation. Moreover, pollutants often constitute a stress for hosts, impacting their immuno-competence (Lafferty and Kuris, 1999). Thus, one direct consequence would be a higher prevalence of parasites due to an increase in hosts susceptibility to infection (Khan, 1990). In addition, many studies showed that infection by parasites increases hosts susceptibility to pollutants in terms of mortality (Brown and Pascoe, 1989, Gismondi et al., 2012a, Gismondi et al., 2012c, Khalil et al., 2014).

Chemical substances can also directly interfere with behavioral changes induced by manipulative parasites. Although the mechanisms through which parasites manipulate their hosts are not yet fully understood, the potential role of neuromodulators has been pointed out in several cases (Adamo, 2002, Perrot-Minnot and Cézilly, 2013). It is then very likely that certain pollutants, especially pharmaceuticals, might interfere with those mechanisms. For instance, gammarids infected by manipulative fish acanthocephalans present an increase in brain serotonin immunoreactivity (Tain et al., 2006). In addition, the experimental injection of serotonin in uninfected gammarids led to several behavioral alterations that are quite similar to those induced by acanthocephalan fish parasites (Perrot-Minnot et al., 2014, Tain et al., 2006). Interestingly, fluoxetine, a reuptake inhibitor of serotonin that is widely prescribed as an anti-depressant, can be found in many natural streams (Kolpin et al., 2002). Guler and Ford (2010) found that exposure to both serotonin and fluoxetine altered phototaxis and geotaxis in marine amphipods, two traits often modified by acanthocephalan parasites. In addition, De Lange et al. (2006) showed that even low concentrations of fluoxetine could affect the activity of freshwater amphipods. Although, to our knowledge, the combined effects of manipulative parasites and drug releases have not been investigated, it is very likely that either the intensity of manipulation (due to cumulative effects) or its outcome in terms of increased susceptibility to predation (due to a homogenization of both infected and uninfected hosts behavior), might be altered.

Behavioral alterations induced by parasites rely on hosts' sensory and locomotor systems, which can also be altered by chemical compounds. For instance, host ability to detect chemical cues signaling the presence of a predator and to respond to them can be disrupted by some manipulative parasites. While rats normally display a natural aversion for cat odor, individuals infected by *Toxoplasma gondii* show no aversion, and sometimes attraction, to odors of certain cats (Berdoy et al., 2000, Kaushik et al., 2014). Amphipods *G. pulex* infected by the acanthocephalan *P. laevis* are also attracted to predator odor (Baldauf et al., 2007, Kaldonski et al., 2007; Perrot-Minnot et al., 2007). Pollutants are very diverse and can have many negative effects, including disruption of hosts' sensory systems, such as chemoreceptive performances (Blaxter and Hallers-Tjabbes, 1992, Tierney and Atema, 1986), and might then interfere with manipulation based on the detection and reaction to chemical cues coming from predators. Moreover, those disruptions are likely to have consequences on the physiology and behavior of both uninfected and infected individuals (Scott and Sloman, 2004, Zala and Penn, 2004). Once again, the interaction between those effects and the alterations induced by manipulative parasites remain to be investigated.

Despite the lack of studies about the effects of pollutants, parasites have received substantial attention from scientists in relation to their ability to accumulate heavy metals such as cadmium and lead. Although the phenomenon is not restricted to manipulative parasites, it has been particularly well documented in adult acanthocephalans (Sures et al., 1999) infecting diverse vertebrate hosts, such as rats (Scheef et al., 2000) or fish (Sures and Taraschewski, 1995). In such host species, harboring parasites might be an advantage in polluted environments, because of the ability of parasites to detoxify host tissues (Thomas et al., 2000). Larval acanthocephalan parasites, on the other hand, can affect the antitoxic response of their intermediate hosts to heavy metals (Gismondi et al., 2012a), often inducing a higher mortality (Brown and Pascoe, 1989). However, this pattern may actually depend on the sex of the host. Indeed, Gismondi et al. (2012b) found that, unlike females, infected male gammarids had both lower cadmium concentrations, and lower mortality compared to uninfected males. In this case, being infected might be, overall, beneficial, despite the increased probability of being predated.

### 3.4 Habitat and resources modifications

Environmental modifications can lead to other types of habitat alterations that are also likely to alter the interaction between hosts and their manipulative parasites. Importantly, habitat alterations might induce changes in the geographical distribution of species, including parasites' hosts and vectors (reviewed in Lafferty and Kuris, 1999).

Apart from effects on hosts' communities, the configuration of hosts' habitats, especially in rivers, can directly impact parasite manipulation or its outcome. For instance, *G. pulex* individuals manipulated by the acanthocephalan *P. laevis* were found to be significantly more predated than uninfected individuals only when refuges were available (Kaldonski et al., 2007). One of the consequences of environmental changes could be a modification in the availability of refuges, notably due to modifications of water levels due to global warming. A decrease in refuge availability is then likely to make manipulation of gammarids ineffective. The alteration of phototaxis in amphipods infected with an acanthocephalan has also been shown to depend on light properties (Benesh et al., 2005, Perrot-Minnot et al., 2012). Considering that phototaxis is one of the most strongly altered behaviors in infected gammarids (Perrot-Minnot et al., 2014), we can expect the light regime in the environment to play a role in the outcome of manipulation. In particular, eutrophication of freshwater bodies induces modifications of light penetration into the water (Van Duin et al., 2001). The same phenomenon is also likely to alter underwater vision, and, hence, reduce the predatory success of final hosts. Thus, if parasite manipulation relies on visual cues to increase the susceptibility of infected hosts to predation, its efficiency might be altered following perturbations of the light regime (but see Perrot-Minnot et al., 2012).

Eutrophication, as well as modifications in any food resources, are also likely to alter host and parasite communities (see Marcogliese, 2001). Host life history traits, such as size or immune capacities, also depend on their diet. On the other hand, host resources are essential for parasites to develop, and many studies found that fewer parasites would develop if their hosts are starving (Logan et al., 2005, Pulkkinen and Ebert, 2004, Seppälä et al., 2008a). In contrary, an increase in host resources might reduce the competition between parasites within hosts, and allow the co-existence of multiple parasites (Dianne et al., 2012, Labaude et al., 2015), leading to modifications in manipulation intensity (see Table 1). Substantial host resources might also lead to the development of larger parasites, and Dianne et al. (2012) highlighted that larger larval acanthocephalans induce deeper modifications in phototactic preferences of their gammarid hosts. The distribution of hosts’ resources can also influence the trophic transmission of parasites. For instance, Luong et al. (2014) found that the availability of alternative food resources for final hosts decreased their infection by trophically-transmitted parasites, as a consequence of reduced predation upon intermediate hosts. In this case, manipulation might, once again, become ineffective. Although their direct effects on manipulation remain to be studied, resources might thus play a role in the interaction between manipulative parasites and their hosts.

## 4 Conclusions and future directions

The examples provided here highlight the importance of the interaction between environmental changes and manipulative parasites. However, most of the studies cited here considered this interaction in single specific contexts. Although simplifications are essential to disentangle the roles of each component, it has to be kept in mind that many of the factors discussed above might occur simultaneously. For instance, ecosystems often face several anthropic disturbances in concert, while only few studies considered such combined effects (e.g. Alonso et al., 2010). On the other hand, a single factor is also likely to affect several protagonists of ecosystems. For instance, we highlighted earlier that fluoxetine might increase predation on exposed prey, by inducing behavioral modifications that are close to those induced by manipulative parasites. However, this increased predatory rate might be balanced by impaired predation success in fish predators exposed to fluoxetine (Gaworecki and Klaine, 2008). We suggest that future studies should adopt a more integrative approach, taking into account multiple components of the systems as well as their interactions. For this, long term studies and field studies might be appropriate tools to bring a better understanding of the complexity underlying the role of manipulative parasites in a changing world. For example, as proposed earlier in this review, we suggest to investigate the combined effects of both parasite-mediated and climate-mediated stresses on the immune system, in order to understand effects on parasite manipulation, and investigate the combined effects of manipulative parasites and contaminant releases on the host's susceptibility to predation. We also propose to explore the effect of global change on several components of systems involving manipulative parasites. For instance, although testing the effect of an increase of temperature on host manipulation is needed, its consequences cannot be understood without also testing the effect of temperature on transmission success, since both the intermediate (manipulated) and the final hosts (predator of the intermediate host) will experience the increase in temperature.

Most of the environmental changes considered here are quite recent, such that adaptive modifications might not be visible yet, leading to a higher consideration from scientists for direct ecological consequences rather than evolutionary ones. However, the intensity or the timing of manipulation are likely to evolve in response to global change. For instance, hosts might suffer from a higher mortality induced by many stressors, such as higher temperatures and pollution. Thomas et al. (2002) suggested that parasites might benefit from adjusting their exploitation strategy depending on the probability of near death of their host. If expected life-span is reduced for every individual host, we might expect an overall better success for parasites which are able to manipulate their hosts sooner and in more efficient ways, allowing a higher probability of transmission to the next host before the death of their intermediate host. Similarly, Lebarbenchon et al. (2008) suggested that parasite strains with different levels of virulence might be selected when environmental conditions affect the survival of infective stages. In the case of manipulative parasites, higher manipulative efforts might be expected as a compensation for the loss of infective stages in those environments. However, the adaptation of manipulative parasites to rapid environmental changes is questionable, as it relies on parameters which have been poorly studied. For example, only a few studies are available on both host and parasite genetic variation (review in Thomas et al., 2011), the raw material for evolutionary adaptation. Therefore, investigations on genetic variation and reaction norms among contrasted environments are necessary to know if responses of manipulative parasites to environmental changes (i) are possible and (ii) result from selection or phenotypic plasticity.

On the other hand, we might expect manipulation to decrease in response to other environmental disturbances. As discussed earlier, harboring parasites accumulating heavy metals could be advantageous for their definitive hosts in a polluted environment, due to the parasites ability to detoxify the host. Those predator hosts might then benefit from feeding specifically on infected preys, whether manipulated or not. Therefore, it could be worth investigating the consequences of benefits associated with detoxification on the manipulation phenomenon to answer the following questions: Are predation behaviors of definitive hosts different between polluted and clean environments? Could manipulation be counter-selected in polluted environments, provided that contamination show some stability in time?

Finally, manipulative parasites deserve more attention in applied sciences. Despite their numerous roles, epidemiologic models keep ignoring their impact on the spread of infectious diseases. In the field of conservation biology, they are also largely overlooked. However, their impact on the success of biological invasions proves that introduced species should be considered along with their parasites in order to make accurate predictions on their probability of establishment success. Thus, apart from invasion problematics, manipulative parasites are also likely to drive the success of reintroductions, for example. In the case of population reinforcement with individuals coming from different geographic locations, the question would arise whether or not those individuals should be relocated with their own parasites, and whether local manipulative parasites are likely to alter those individuals in a similar ways, thus not disturbing the role of reintroduced animals in the ecosystem. Manipulative parasites, although they could be a burden in conservation biology, are also likely to become helpful tools. In a recent paper, Tompkins and Veltman (2015) showed that *T. gondii* could be used to improve vertebrate pest control. This parasite induces several behavioral modifications in its rat host, among which a decreased neophobia and an increased activity (Webster, 1994, Webster et al., 1994). Rats constitute a highly invasive species in New Zealand, and a substantial threat for indigenous species. Trapping is often used to control rats’ populations, but the natural neophobia of rats renders them hard to capture. Tompkins and Veltman (2015) reported that infection by *T. gondii* widely increases the trapability of rats, and that infection would reduce the trapping efforts required to maintain rat population under a threshold for conservation benefit. We follow them in considering that other manipulative parasite species might be of interest for ecosystems and population management.

## Figures and Tables

**Table 1:** Parameters affecting the intensity of parasite manipulation.

Parameter	Host	Parasite	Trait modified	Reference
*Parameters intrinsic to the parasite*				
Age/stage of the parasite	Amphipod	Acanthocephalan	Phototaxis	Franceschi et al., 2010a, Franceschi et al., 2008
Amphipod	Acanthocephalan	Refuge use	Dianne et al., 2011
Isopod	Acanthocephalan	Mating behavior	Sparkes et al., 2006
Insect	Protozoan	Host-seeking	Koella et al., 2002
Insect	Nematomorph	Jumping into water	Sanchez et al., 2008
Rodent	Nematode	Activity	Dolinsky et al., 1985
Fish	Trematode	Aggressiveness	Mikheev et al., 2010
Parasite sibship	Amphipod	Acanthocephalan	Phototaxis	Franceschi et al., 2010a
Parasite population	Amphipod	Acanthocephalan	Phototaxis	Franceschi et al., 2010b, Labaude et al., 2015
Genetic strain	Amphipod	Acanthocephalan	Phototaxis	Perrot-Minnot, 2004
Parasite sex	Isopod	Acanthocephalan	Colouration	Benesh et al., 2009a, Benesh et al., 2009b
Parasite size	Amphipod	Acanthocephalan	Phototaxis	Dianne et al., 2012
Fish	Cestode	Demelanization	Ness and Foster, 1999
*Parameters intrinsic to the host*				
Host size	Isopod	Acanthocephalan	Colouration	Benesh et al., 2009a, Benesh et al., 2009b
Host weight	Amphipod	Acanthocephalan	Activity	Dianne et al., 2014
Host age	Fish	Trematode	Motionless	Poulin, 1993
*Parameters relative to the infection*				
Parasites total volume	Isopod	Acanthocephalan	Colouration	Benesh et al., 2009a, Benesh et al., 2009b
Parasite load	Amphipod	Acanthocephalan	Phototaxis	Franceschi et al., 2008
Fish	Trematode	Motionless	Poulin, 1993
Mollusc	Trematode	Burrowing ability	Mouritsen and Poulin, 2003
Multi-infection with	Amphipod	Acanthocephalan	Phototaxis	Dianne et al., 2010
Different stages	Copepod	Cestode	Activity	Hafer and Milinski, 2015
Multi-infection with different parasite species	Amphipod	Acanthocephalan, microsporidia	Geotaxis	Haine et al., 2005
Amphipod	Acanthocephalan	Vertical distribution	Cézilly et al., 2000
Mollusc	Trematodes	Distribution	Miura and Chiba, 2007
Mollusc	Trematodes	Shell size	Miura and Chiba, 2007
